# Rapid diagnosis of *Propionibacterium acnes* infection in patient with hyperpyrexia after hematopoietic stem cell transplantation by next-generation sequencing: a case report

**DOI:** 10.1186/s12879-015-1306-0

**Published:** 2016-01-08

**Authors:** Mingzhi Ye, Wei Wei, Zhikai Yang, Yingzhen Li, Shaomin Cheng, Kang Wang, Tianliangwen Zhou, Jingmeng Sun, Sha Liu, Na Ni, Hui Jiang, Hua Jiang

**Affiliations:** 1 BGI-Guangdong, BGI-Shenzhen, Guangzhou, 510006 China; 2 BGI-Guangzhou, Guangzhou Key Laboratory of Cancer Trans-Omics Research, Guangzhou, China; 3 Hematopoietic Stem Cell Transplant Center, Guangzhou Women and Children Medical Center, Guangzhou, 510000 China; 4 BGI-Shenzhen, Shenzhen, 518083 China

**Keywords:** Propionibacterium acnes, Hyperpyrexia, Hematopoietic stem cell transplantation, Next generation sequencing, Diagnosis

## Abstract

**Background:**

The rapid determination of pathogenic agent is very important to clinician for guiding their clinical medication. However, current diagnostic methods are of limitation in many aspects, such as detecting range, time-consuming, specificity and sensitivity. In this report, we apply our new-developing pathogen detection method to clarify that *Propionibacterium acnes* is the causative agent of a two-year-old boy with juvenile myelomonocytic leukemia presenting clinical symptoms including serious rash and hyperpyrexia while traditional clinical methods of diagnosis fail to detect the pathogenic agent and multiple antimicrobial drugs are almost ineffective *Propionibacterium acnes* is confirmed to be the infectious agent by quantitative real-time polymerase chain reaction.

**Case presentation:**

After haploidentical hematopoietic stem cell transplantation, a two-year-old boy with juvenile myelomonocytic leukemia presented to a pediatrist in a medical facility with hyperpyrexia and red skin rash which later changed to black skin rash all over his body. Traditional diagnostic assays were unrevealing, and several routine antimicrobial treatments were ineffective, including the vancomycin, meropenem, tobramycin, cefepime and rifampin. In this case, pediatrist resorted to the next-generation sequencing technology for uncovering potential pathogens so as to direct their use of specific drugs against pathogenic bacteria. Therefore, based on the BGISEQ100 (Ion Proton System) which performed sequencing-by-synthesis, with electrochemical detection of synthesis, and each such reaction coupled to its own sensor, which are in turn organized into a massively parallel sensor array on a complementary metal-oxidesemiconductor chip, we detect and identify the potential pathogens. As a result, we detected a significantly higher abundance of skin bacteria *Propionibacterium acnes* in patient’s blood than controls. It had been reported that patients infected by *Propionibacterium acnes* almost always had history of immunodeficiency, trauma or surgery. Considering this possible cause, antimicrobial treatment was adjusted to target this rare opportunistic pathogen. Fever and black skin rashes were rapidly reduced after administrating specific drugs against *Propionibacterium acnes*.

**Conclusion:**

This case showed our new-developing pathogen detection method was a powerful tool in assisting clinical diagnosis and treatment. And it should be paid more attention to *Propionibacterium acnes* infection in clinical cases.

## Background

*Propionibacterium acnes (P. acnes)* is a skin commensal bacterium. On rare occasions, it can cause serious postoperative complications, such as infective endocarditis, thrombophlebitis, and acute suppurative pericarditis, especially in immunodeficiency patients who are highly susceptible to pathogenic microorganisms. And these infections often run an acute course due to the weak or late inflammatory response [1], but their clinical symptoms are usually atypical, making them very difficult to diagnose. Till now, there have been various pathogen-detecting methods applied in clinic, such as isolation and culture, serological test, specific polymerase chain reaction (PCR) and its derivatives and so on, but all these methods are almost always based on the known sequences or components and usually target one or several major known pathogens, presenting serious limitations [2]. As the development of sequencing technology with its cost continually falling, next-generation sequencing (NGS) has become an attractive tool for broad-based pathogen discovery. NGS have strong potential to detect and identify almost varieties of microorganisms, including known and unknown. In 2008, Palacios G et al. identified a new virus from three patients who received visceral-organ transplants from a single donor by high-throughput sequencing, showing a powerful tool for discovery of new pathogens [3]. In 2013, a 14-year-old boy was enrolled for pathogen detection by use of NGS because of the failure of traditional diagnostic assays, resulting ultimately in a favorable outcome [4],which indicated that the pathogen detection method based on NGS could be useful in clinical cases, Recently, a semiconducting sequencing platform (BGISEQ100) has been improved greatly, possessing some remarkable technique features, such as fast (sequencing within 2–3 h), flexible (flexible scaled chips for different throughput needs) and high accuracy (99.97 %). Here, we utilize this platform to develop a pathogen detection method for discovering the potential pathogens of unknown infection, successfully identifying an opportunistic pathogen (*P. acnes*) that may be the major cause of serious infection of a two-year-old boy. Our result shows a reliable method for detecting potential pathogens of unknown infection in HSCT patients, indicating its strong application values in clinic.

## Case presentation

**Fig. 1:**
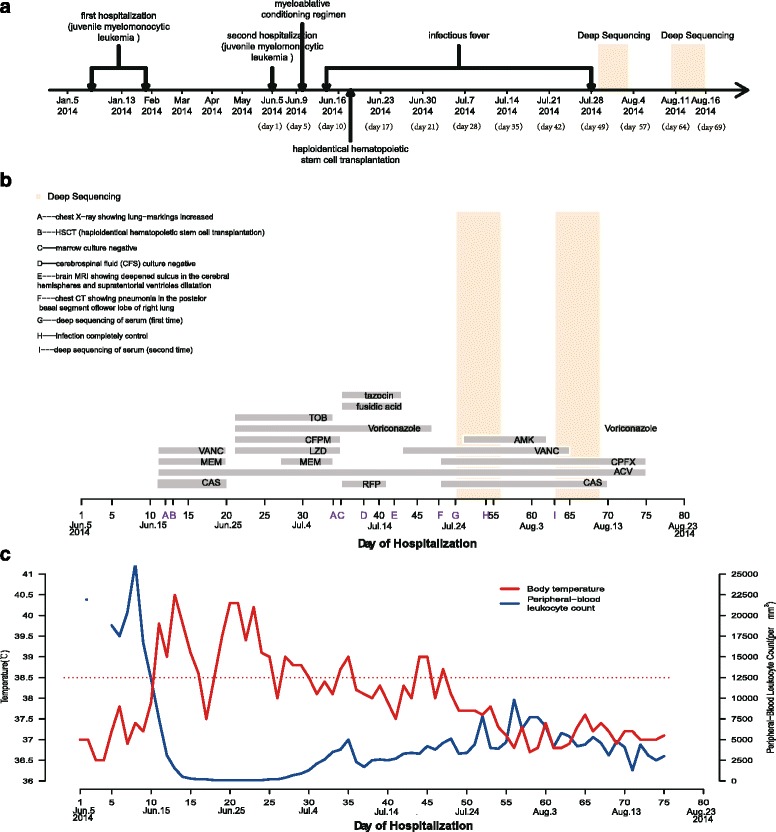
Clinical course (Panel **a**), antibiotic regimens (Panel **b**), body temperature and leukocyte count (Panel **c**) of the 2-year-old patient with hyperpyrexia

On January 5th, 2014, a 2-year-old boy with JMML was presented to the pediatric hematology and oncology department for abnormal hemogram lasting for two months. Then, he was admitted to the hospital and discharged 8 days later after completing the Human Leukocyte Antigens (HLA) matching (Fig. 1a). Subsequently, he continued with chemotherapy and outpatient medications, including mercaptopurine, prednisone, and 13-cis-retinoid acid (isotretinoin).

**Fig. 2:**
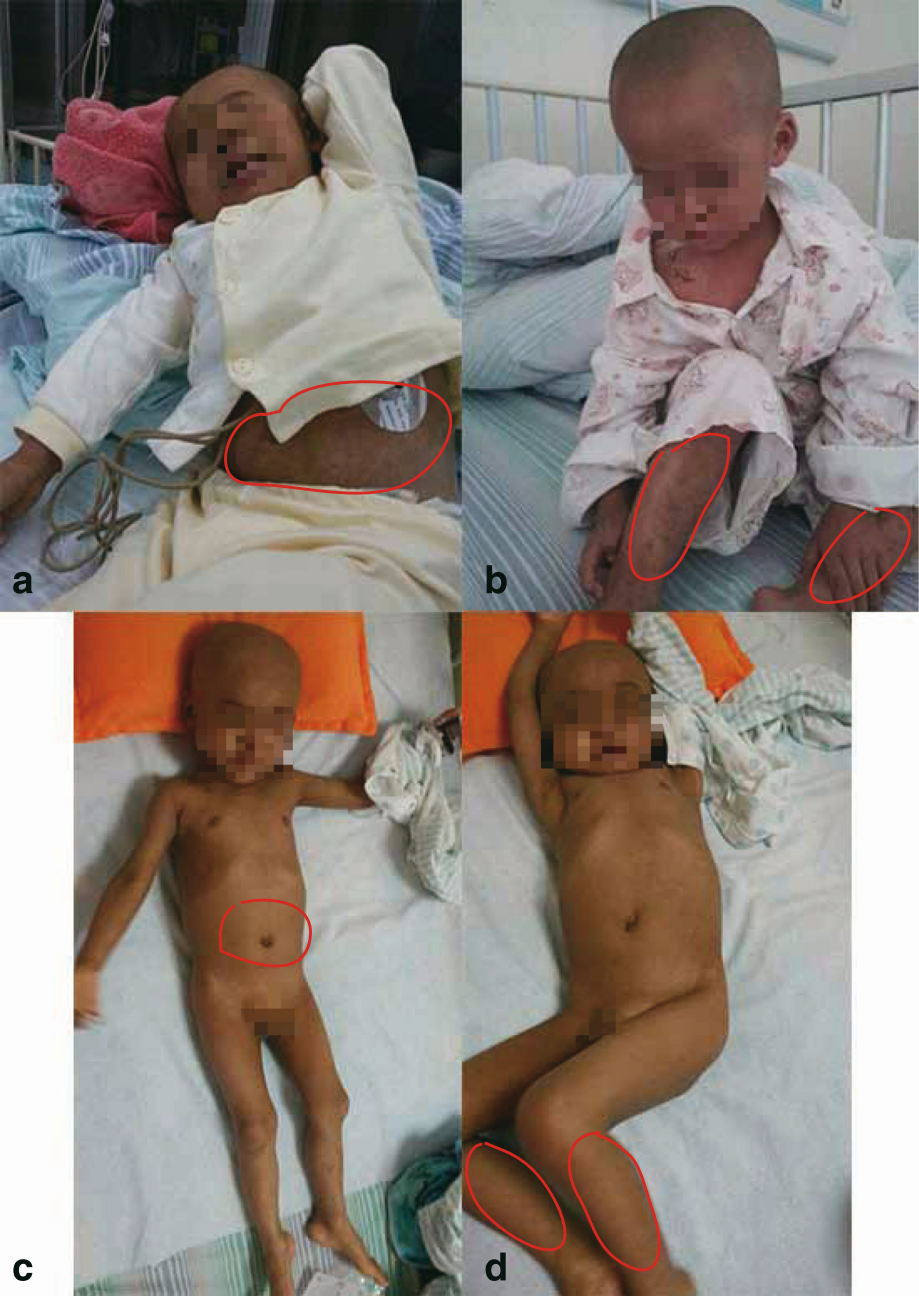
The situation of skin rashes on patient’s body in two time-points (pre- and post-treatment). The patient presented hyperpyrexia with black rash all over his body in the first detection (Panels **a**, **b**). The patient’s body temperature returned to normal with the rash fading in the second detection (Panels **c**, **d**)

On June 5th, 2014, the patient returned to the hospital for preparation before HSCT. His vital signs were normal, and physical examination was unremarkable except some discrete old rashes on skin (Fig. 2). The result of hospital laboratory examinations was also normal. Since June 10th, patient started to receive a myeloablative conditioning regimen [busulfan (BU) 1.2 mg/kg iv. q6h for 4 days, cyclophosphamide (CTX) 50 mg/kg iv. qd for 4 days and anti-thymocyte globulin (ATG) 3.3 mg/kg iv. qd for 3 days]. However, the patient suffered from fever (up to 39.8 °C) with mild cough and running nose since June 15th, indicating respiratory infection. Subsequently, clinical examinations were performed to analyze the possible cause. The blood/marrow culture were negative. The number of his peripheral white blood cells and neutrophils was 7500 and 2450 per cubic millimeter, respectively. The C-reactive protein (CRP) was above 200 mg per liter (normal range, 0 to 5 mg per liter). The procalcitonin (PCT) was 0.15 ng per milliliter (normal range, 0 to 0.1 ng per milliliter). The G test which was target for broad spectrum dectection of fungal infection was 20.6 pg per milliliter (normal range, 0 to 20 pg per milliliter). All these results from assays of serum liver-enzyme, usea nitrogen, creatinine, electrolyte and pathogen specific antigen were within range of normal value. However, the result of chest X-ray showed increased lung-markings. Consequently, the patient was treated with meropenenm (MEM) targeted for gram-negative and other refractory bacteria, and vancomycin (VANC) targeted for gram-positive cocci. Meanwhile, antifungal agent caspofungin acetate (CAS) and antiviral agent acyclovir (ACV) were also administered as prophylaxis (Fig. 1b).

The patient underwent the cord blood transplantation and HSCT (the donor was his mother) on June 20th and 21th, respectively. From June 15th to June 24th, patient’s body temperature dropped to 38.5 °C from 40.5 °C, then rose to 40.3 °C again (Fig. 1c). Meanwhile, CRP dropped to 8.5 mg per liter (normal range, 0 to 5 mg per liter).

Although treatments had been performed regularly, his symptoms of fever, chills and cough still didn’t lighten. Physical examination showed some previous discrete rashes and coarse lung sounds. CRP was 119.2 mg per liter (normal range, 0 to 5 mg per liter). MEM and VANC almost had no effect on the clinical symptoms. Then, his primary care physician decided to switch drugs to linezolid (LZD) against gram-positive cocci, tobramycin (TOB) against gram-negative bacteria for a week, and voriconazole for fungal prophylaxis (Fig. 1b).

**Fig. 3:**
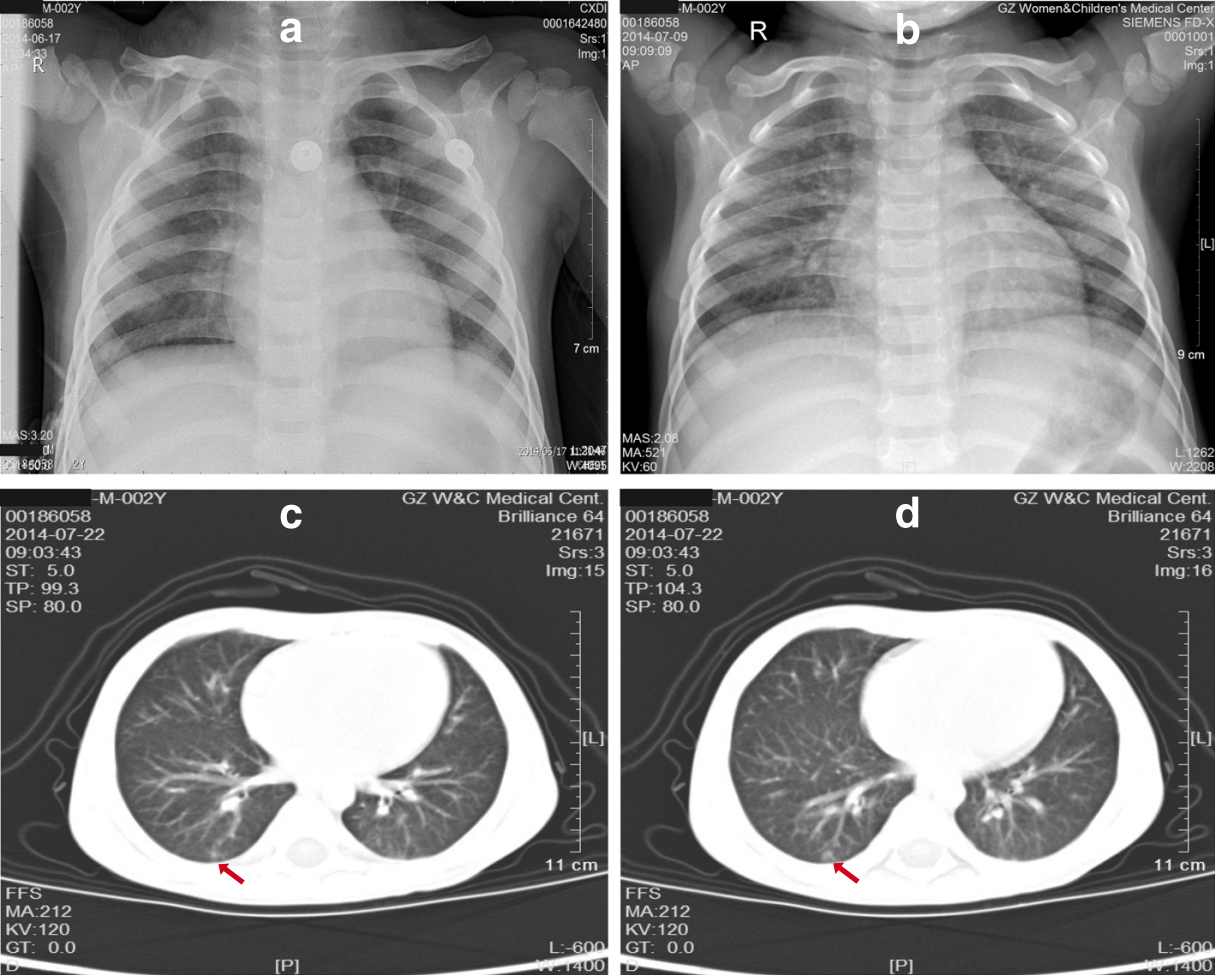
The results of chest X-ray, CT of patient. The chest X-ray images showed slightly increased lung-markings from Jun 17th to July 9th (Panels **a**, **b**), and images of chest CT revealed pneumonia in the posterior basal segment oflower lobe of right lung on July 22nd (Panels **c**, **d**)

During this period, patient also experienced anemia and thrombocytopenia occasionally. After transfusion of washed red blood cells and platelets for skin bleeding, the number of his hemoglobin and platelets returned to normal. During the next two weeks, he still presented with fever, mild expectoration and some discrete old rashes, suggesting the possibility of *tuberculosis bacillus* infection. Considering the medication safety, the doctor stopped using TOB and LZD, and changed to use the rifampicin (RFP) and fusidic acid against gram-positive bacteria and tazocin against drug-resistance gram-negative bacteria (Fig. 1b). On July 13th, lumber puncture was performed to collect cerebrospinal fluid (CSF) for *tubercle bacillus* detection, but no positive results were obtained by culture and PCR based methods. After a week, the computed tomography (CT) of chest showed pneumonia in the posterior basal segment of the lower lobe of right lung (Fig. 3c/d), and CRP became 0.2 mg per liter (normal range, 0 to 5 mg per liter), but his body temperature still fluctuated between 37.7 °C and 39 °C. Finally, we got the informed consent of patient’s mother on behalf of the patient for detecting potential pathogens based on NGS technology (Fig. 1a).

Within 43 h after collecting patient’s blood, we finished the detection of pathogens, identifying 22,123 (out of 2,602,891) sequence reads (0.8499 %) uniquely corresponding to *Propionibacterium acnes* (*P. acnes, G* ^*+*^) genome. *P. acnes* accounted for a very high proportion in detectable microorganisms and possessed a high coverage of genome, which was also significantly higher than control (Fig. 5). This result was re-confirmed by the NGS detection and species specific real-time PCR (RT-PCR). Based on the result, the primary care physician turned to adopt specific drugs for treatment of *P. acnes* infection immediately, including VANC, ciprofloxacin (CPFX) and amikacin (AMK) against gram-positive bacteria, CAS and ACV for prophylaxis of fungal and virus infections.

Over the next 5 days, the patient gradually recovered with his body temperature returning to normal range and his old rash fading (Fig. 2c/d). However, other complications still persisted, such as the graft versus host disease (GVHD).

## Methods

**Fig. 4:**
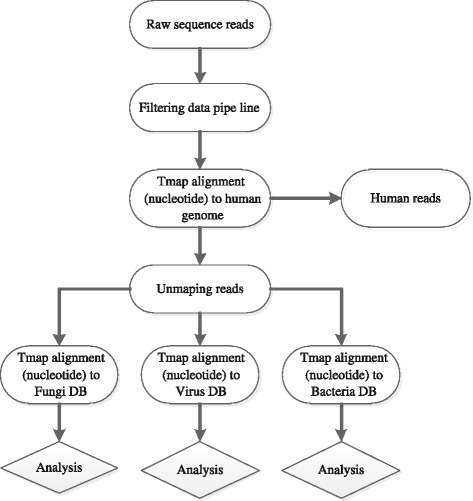
The schematic overview of BGI analysis pipeline

The NGS-based pathogen detection of patient’s blood sample was approved by his parents and primary care physician. Blood samples were processed in a medical laboratory according to the Ion Torrent next-generation sequencing assay manual (BGISEQ100). The general detection process was: 1) 200 μl plasma was used to extract nucleic acid for cDNA libraries; 2) sequencing was performed by BGISEQ100 after library was validated by 2100 Bioanalyzer system (Agilent Technologies, Inc.) and RT-PCR. Sequence reads were classified according to their origin by a bioinformatics pipeline developed by BGI (Fig. 4). The bioinformatics process mainly included the following steps: 1) host reads were subtracted; 2) remained reads were aligned to reference database, composed of multiple public sequence resources of bacteria, viruses and fungi. And the reference database was constructed according to the following steps:

1. downloaded all complete genome sequences of Bacteria, Virus from NCBI ftp site ftp://ftp.ncbi.nih.gov/genomes/;
2. removed the plasmid sequence and any other non-human-related sequence;
3. kept only one best genome sequence representing every species based on the assembly result;
4. built the alignment index of reference sequence for classing sequence reads.

In order to validate the result of NGS-based detection,specific primers were designed to detect the *P. acnes* in samples by RT-PCR. In order to accurately compare the relative abundance difference of *P.acnes* among different samples, human beta-actin was used as the internal reference. The designed primers were listed as:

KPA171202 of *P.acnes* (PA-F:5′-GCGTGAGTGACGGTAATGGGTA-3′ and PA-R:5′-TTCCG ACGCGATCAACCA-3′), and Beta-actin of human (βA-F:5′-AACGGCTACCACATCCAAGG-3′ and βA-R:5′-ACCAGACTTGCCCTCCAATG-3′).

## Results

### Rapid identification of *P. acnes* sequences in blood plasma

**Fig. 5:**
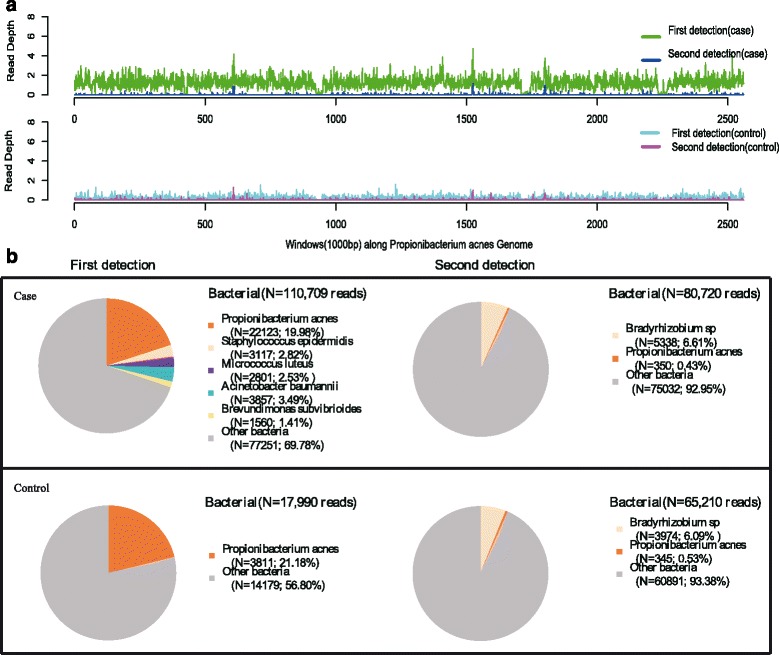
Diagnosis of *P. acnes* infection by the NGS-based method: mapping of *P. acnes *reads (Panel **a**) and sequences in blood of two detections (Panel **b**)

In detection of each sample, we selected a control sample from a non-infected patient in the same ward to do the same detection.In all of the four samples, the RNA of each sample was extracted to construct the cDNA library for sequencing. As the sequencing result, the number of reads from cDNA library of patient’s plasma was 2,602,891, and that of control was 4,294,544. As the result of pathogen detection, *P. acnes* was identified as the most predominant pathogen taking up 0.85 % (22,123 out of 2,602,891) of total sequence reads, 19.98 % of total bacterial reads and 65 % coverage of *P. acnes* genome in the first detection but reduced to 0.0048 % (350 out of 7,250,976 reads) of total sequence reads, 0.43 % of bacterial reads and 1.5 % coverage of *P. acnes* genome in the second detection after specific drug treatments, approximate to the number of *P. acnes* reads in control (Fig. 5 and Table 1).

**Table 1:** The basic situation of *P. acnes* in twice NGS-based detections

Item	Reads number of *P. acnes*	Proportion of *P. acnes* reads in total reads	Coverage of *P. acnes* genome	Depth of *P. acnes* genome
First detection (case)	22,123	0.8499 %	65.00 %	1.9
First detection (control)	3,811	0.0887 %	17.00 %	1.2
Second detection (case)	350	0.0048 %	1.50 %	1.2
Second detection (control)	345	0.0048 %	1.40 %	1.3

### Confirmatory testing for *P. acnes*

**Fig. 6:**
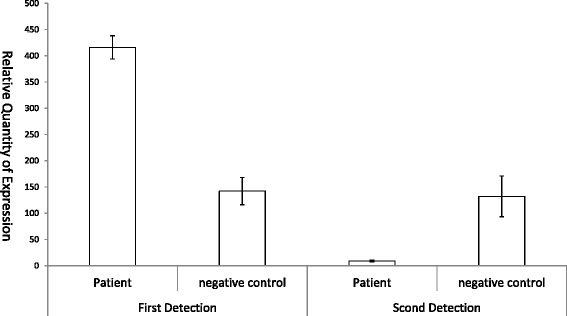
Validation of P. acnes in patient’s plasma by RT-PCR

Identification of *P. acnes* in patient’s plasma was confirmed by RT-PCR targeted the specific gene KPA171202.As the RT-PCR result, it was no statistically significant (*P* = 0.56, Student’s *t*-test) of the negative control in two detections, but the patient was higher (*P* = 0.0056) than negative in the first detection and it was significantly reduced (*P* = 0.00094) in the second detection (Fig. 6), which showed a high consistency with the NGS-based result.

## Discussion

*P. acnes* is a typical gram positive, anaerobic bacterium, which belongs to the normal skin microbiota, usually colonize skin surface and closely link with skin acne. However, its potential role in clinical infection is often underestimated due to its low virulence [5]. Furthermore, *P. acnes* is rarely diagnosed to be the major pathogen leading to serious infection not only for its pathogenicity but also methology. In aspect of clinical pathogen detection, the most common method is the culture-based method, but it possesses very poor sensitivity and accuracy and often needs to consume a large amount of time (>7d) to grow microbe for analysis. Other traditional detection methods are also becoming more and more of limitation due to continual variation of microorganisms, emergence of new pathogens and flaw of method itself such as low accuracy, poor specificity and so on. Therefore, it is highly necessary to develop or utilize new detection methods to cope with these limitations. The NGS-based pathogen detection is a new-developing method for scanning the microbial sequences in clinical samples, by which we can easily identify potential pathogens for performing specific antimicrobial treatment. In clinic, *P. acnes* is gradually recognized as an important factor of human opportunistic infection as the number of clinical case appears to be on the rise [6–11]. However, atypical symptoms of *P. acnes* infections often lead to the confusion with postoperative complications, such as prosthetic joint infection, pacemaker endocarditis, and implant-associated infections [6]. In this case, although his antibody of HIV was always negative, with nutritional support therapy against malnutrition, it is highly possible that this patient is infected by the opportunistic *P. acnes* resided in the skin dermis because of his defective immune system [12]. The significant differences of read number and genomic coverage of *P. acnes* between patient and control and pre- and post-treatment strongly indicate the infection of *P. acnes.*

Before confirming infection of *P. acnes*, Pediatricians had to choose various drugs targeting viruses, fungi, and bacteria (G^+^ bacterium, G^−^ bacterium, bacillus and coccus) base on the principles of minimum damage to patients and universal prescription drug coverage. However, *P. acnes* is able to form biofilm, which renders it resistant to most antibiotics [13, 14], such as daptomycin and rifampin [14, 15]. The recently published report suggests that the combination of daptomycin and rifampin, followed by levofloxacin and rifampin, might be a reasonable treatment for *P. acnes* infection [15]. Subsequently, the primary care doctor administrates drugs according to the reported dosage regimen, attaining an effective treatment. Additionally, biofilm formation is essential for resistance of some bacteria to drug. *P. acnes* has been shown to be able to form biofilm both *in vitro* and *in vivo* [16], which also highlights the important influence of biofilm in treatment of *P. acnes* infection [17].

## Conclusion

In summary, our NGS coupled with an efficient bioinformatics pipeline successfully identified an opportunistic pathogen responsible for an infection of unknown origin, which eluded conventional assays in clinic, powerfully assisting doctors in selecting the most targeted and effective treatment for patients. The present case study also has important clinical implications, *P. acnes* infections may continue to increase in the patients with immuno-compromise in the future, and an optimal antimicrobial regimen needs to be defined.

## Consent

Written informed consent was obtained from the patient’s parent for publication of this case report. A copy of the written consent is available for review by the Editor of this journal.

## Abbreviations

P. acnes: Propionibacterium acnes
PCR: Polymerase chain reaction
NGS: Next-generation sequencing
HLA: Human Leukocyte Antigens
HSCT: Haploidentical hematopoietic stem cell transplantation
JMML: Juvenile myelomonocytic leukemia
BU: Busulfan
CTX: Cyclophosphamide
ATG: Anti-thymocyte globulin
CRP: C-reactive protein
PCT: Procalcitonin
MEM: Meropenenm
VANC: Vancomycin
ACV: Antiviral agent acyclovir
LZD: Linezolid
TOB: Tobramycin
RFP: Rifampicin
CSF: Cerebrospinal fluid
CT: Computed tomography
RT-PCR: Real-time PCR
CPFX: Ciprofloxacin
AMK: Amikacin
CAS: Caspofungin acetate
GVHD: Graft versus host disease

## References

[1] Picard C, Casanova JL, Puel A (2011). Infectious diseases in patients with IRAK-4, MyD88, NEMO, or I_K_Bαdeficiency. Clin Microbiol Rev.

[2] Chiu CY (2013). Viral pathogen discovery. Curr Opin Microbiol.

[3] Palacios G, Druce J, Du L, Tran T, Birch C, Briese T (2008). A new arenavirus in a cluster of fatal transplant-associated diseases. N Engl J Med.

[4] Wilson MR, Naccache SN, Samayoa E, Biagtan M, Bashir H, Yu G (2014). Actionable Diagnosis of Neuroleptospirosis by Next-Generation Sequencing. N Engl J Med..

[5] McDowell A, Patrick S, Dongyou L (2011). “Propionibacterium,” in Molecular Detection of Human Bacterial Pathogens.

[6] Zappe B, Graf S, Ochsner PE, Zimmerli W, Sendi P (2008). Propionibacterium spp. in prosthetic joint infections: a diagnostic challenge. Arch Orthop Trauma Surg.

[7] Clayton JJ, Baig W, Reynolds GW, Sandoe JA (2006). Endocarditis caused by Propionibacterium species: a report of three cases and a review of clinical features and diagnostic difficulties. J Med Microbiol.

[8] Arnell K, Cesarini K, Lagerqvist-Widh A, Wester T, Sjölin J (2008). Cerebrospinal fluid shunt infections in children over a 13-year period: anaerobic cultures and comparison of clinical signs of infection with Propionibacterium acnes and with other bacteria. J Neurosurg Pediatr.

[9] Perry A, Lambert P (2011). Propionibacterium acnes: infection beyond the skin. Expert Rev Anti Infect Ther.

[10] Levy O, Iyer S, Atoun E, Peter N, Hous N, Cash D (2013). Propionibacterium acnes: an underestimated etiology in the pathogenesis of osteoarthritis?. J Shoulder Elbow Surg..

[11] Santo KR, Franceschi V, Campos AC, Monteiro TS, Barbosa GI, Dantas A (2014). Pacemaker Endocarditis Caused by Propionibacterium acnes in an Adult Patient with Ebstein’s Anomaly: A Report of a Rare Case. Heart Lung Cric..

[12] Lee MJ, Pottinger PS, Butler-Wu S, Bumgarner RE, Russ SM, Matsen FA (2014). Propionibacterium persists in the skin despite standard surgical preparation. J Bone Joint Surg Am.

[13] Kurz M, Kaufmann BA, Baddour LM, Widmer AF (2014). Propionibacterium acnes prosthetic valve endocarditis with abscess formation: a case report. BMC Infect Dis.

[14] Achermann Y, Goldstein EJ, Coenye T, Shirtliff ME (2014). Propionibacterium acnes: from commensal to opportunistic biofilm-associated implant pathogen. Clin Microbiol Rev.

[15] Furustrand Tafin U, Corvec S, Betrisey B, Zimmerli W, Trampuz A (2012). Role of rifampin against Propionibacterium acnes biofilm in vitro and in an experimental foreign-body infection model. Antimicrob Agents Chemother.

[16] Ramage G, Tunney MM, Patrick S, Gorman SP, Nixon JR (2003). Formation of Propionibacterium acnes biofilms on orthopaedic biomaterials and their susceptibility to antimicrobials. Biomaterials.

[17] Holmberg A, Lood R, Mörgelin M, Söderquist B, Holst E, Collin M, et al. Biofilm formation by Propionibacterium acnes is a characteristic of invasive isolates. Clin Microbiol Infect. 2009;15:787–95. 10.1111/j.1469-0691.2009.02747.x 19392888

